# Key Determinants of Immediate Postoperative Pain, Nausea, and Vomiting in Orthognathic Surgery: Insights From a Retrospective Cohort Study

**DOI:** 10.7759/cureus.72806

**Published:** 2024-10-31

**Authors:** Shogo Kikuta, Sho Imai, Nodoka Nagae, Katsuhisa Matsuo, Kiyosato Hino, Yushi Abe, Jingo Kusukawa

**Affiliations:** 1 Dental and Oral Medical Center, Kurume University School of Medicine, Kurume, JPN

**Keywords:** anesthesia, orthognathic surgery, postoperative nausea and vomiting, postoperative pain, related factor

## Abstract

Introduction

Orthognathic surgery frequently leads to immediate postoperative pain (IPP) and postoperative nausea and vomiting (PONV), impacting patient recovery and satisfaction. This study aims to identify key determinants contributing to IPP and PONV following orthognathic surgery.

Methods

A retrospective cohort study was conducted involving patients who underwent orthognathic surgery at Kurume University Hospital between January 2020 and December 2023. Patients were divided into two groups: "mandible alone" and "bimaxillary." Independent variables, including patient-related, surgery-related, and anesthesia-related variables, were evaluated against IPP and PONV using multivariate logistic regression analysis.

Results

The study enrolled 181 patients who underwent orthognathic surgery with an average age of 26.6 ± 8.1 years (range: 16-54 years), of which 139 patients (76.8%) were women. Multivariate analysis identified low body mass index (BMI) as a common significant related factor for both IPP (adjusted odds ratio {OR}, 3.70; P* *= 0.0034) and PONV (adjusted OR, 2.80; P = 0.022). Inhalation anesthesia was significantly associated with IPP (adjusted OR, 9.07; P = 0.049), while higher blood loss and increased local anesthesia volumes were associated with PONV (adjusted OR, 2.82 and 3.19; P = 0.005 and 0.016, respectively).

Conclusion

BMI, total blood loss, anesthesia method, and the total amount of local anesthesia may be significant related factors of postoperative complications following orthognathic surgery. These findings can inform preoperative assessments and anesthesia management to improve patient outcomes.

## Introduction

Orthognathic surgery is a common procedure for correcting dentofacial deformities, often resulting in significant improvements in both aesthetic and functional outcomes. Sufficient patient satisfaction is essential to the success of orthognathic surgery [1-4]. However, complications such as immediate postoperative pain (IPP) and postoperative nausea and vomiting (PONV) often occur after orthognathic surgery and can significantly impact patient recovery and satisfaction. Surgical stress following orthognathic surgery can induce moderate to severe postoperative pain and swelling [5-8]. On the other hand, PONV is among the most common complications following surgery under general anesthesia. Patients undergoing orthognathic surgery are particularly susceptible, with a systematic review indicating incidences of postoperative nausea (PON) and postoperative vomiting (POV) as high as 53.5% and 73.3%, respectively [9]. Additionally, PONV can be a potential risk factor for airway obstruction, especially in patients after orthognathic surgery, followed by intermaxillary fixation. These postoperative complications are influenced by various factors, including gender, age, the type of surgery (mandibular-alone surgery or bimaxillary surgery), surgical time, anesthesia factors, and individual patient characteristics [4,5,9]. To date, only one study has examined the relationship between postoperative pain and PONV following orthognathic surgery, and no study has comprehensively analyzed the interplay of patient-related, surgery-related, and anesthesia-related factors. This study aims to clarify the potential related factors for IPP and PONV, which are frequently observed after orthognathic surgery.

## Materials and methods

Study design and patients

This retrospective cohort study enrolled patients who underwent orthognathic surgery at the Dental and Oral Medical Center, Kurume University Hospital, between January 2020 and December 2023. All enrolled cases were classified into the "mandible alone" group, consisting of bilateral sagittal split ramus osteotomy (BSSRO) and BSSRO with genioplasty (BSSRO + Gen), and the "bimaxillary" group, consisting of Le Fort I osteotomy with genioplasty (Le Fort I + Gen), Le Fort I osteotomy with BSSRO (Le Fort I + BSSRO), and Le Fort I osteotomy with BSSRO and genioplasty (Le Fort I + BSSRO + Gen). The exclusion criteria included a patient with congenital craniofacial deformity, previous facial surgeries, preoperative facial trauma, systemic diseases, sequelae of neuromuscular disorders, or missing essential medical records in the present study. Three oral and maxillofacial surgeons with over 10 years of experience performed all orthognathic surgeries. All patients had a throat pack placed in the pharyngeal cavity before surgery to prevent the aspiration of surgical fluids, and 2% lidocaine with 1:80,000 epinephrine was used as local anesthesia. Remifentanil and fentanyl were administered intraoperatively in all cases. Prior to emergence from anesthesia, all patients received intravenous acetaminophen for postoperative analgesia, with the dosage adjusted according to body weight. Adults weighing less than 50 kg were administered 15 mg per kilogram of body weight, while others received 1,000 mg. All patients who underwent Le Fort I osteotomy had a nasogastric tube placed overnight, which was removed the next day. All patients who underwent BSSRO had a suction drain inserted within the wound, which was removed on postoperative day 2 or 3. Rigid fixation was applied in all cases to stabilize the osteotomy sites, and intermaxillary elastics or wires were used for postoperative traction in all patients. All enrolled patients were evaluated for IPP and PONV postoperatively. The study was approved by the Ethics Committee of Kurume University (approval number: 23215) and was conducted in full compliance with the ethical standards outlined in the Declaration of Helsinki (64th World Medical Association (WMA) General Assembly, Fortaleza, Brazil, October 2013).

Immediate postoperative pain (IPP)

IPP was defined as pain immediately after returning to the ward and was assessed using the numeric rating scale (NRS) [10]. The NRS is an 11-point scale ranging from 0 to 10, where 0 indicates no pain, 1-3 indicates mild pain, 4-6 indicates moderate pain, and 7-10 indicates severe pain. The dependent variable was categorized into two groups: the "no pain to mild pain" and "moderate to severe pain" groups. The independent variables included patient-related factors (gender, age, and body mass index {BMI}), surgery-related factors (PONV, the type of surgical procedure, operation time, and total blood loss), and anesthesia-related factors (anesthesia method, the total amount of local anesthetics, the total amount of fentanyl, and the total amount of remifentanil) [4,5,11].

Postoperative nausea and vomiting (PONV)

The presence or absence of PONV after returning to the ward is assessed based on medical records documenting vomiting or the administration of antiemetics for nausea. The dependent variable was the presence or absence of PONV. The independent variables included patient-related factors (gender, age, BMI, and smoking), surgery-related factors (the type of surgical procedure, operation time, total blood loss, and IPP), and anesthesia-related factors (anesthesia method, the total amount of local anesthesia, the total amount of fentanyl, and the total amount of remifentanil) [12-20].

Statistical analysis

All data were analyzed using JMP® Pro 17.0.0 (SAS Institute, Cary, NC). Quantitative data are presented as means ± standard deviations. Two-tailed unpaired Student's t-test, Fisher's exact test, and Pearson's chi-square test were used for data comparison. Statistical significance was set at a P-value of <0.05. Cutoff values for each independent variable, especially quantitative data, were determined using receiver operating characteristic (ROC) curves with IPP and PONV as the outcome measures. To identify predictors of IPP and PONV following orthognathic surgery, a univariate binary logistic regression analysis was first conducted to calculate crude odds ratios (ORs) and 95% confidence intervals (CIs). This was followed by a multivariate binary logistic regression to determine adjusted ORs with 95% CIs. The dependent variables were IPP and PONV, while the independent variables included those with a P-value of <0.1 in the univariate analysis. Additionally, known factors associated with IPP and PONV, based on prior literature, were included as independent variables [4,5,11-20]. Due to sample size limitations, it was not possible to adjust for all potential confounding factors.

## Results

Patient characteristics

A total of 181 patients who underwent orthognathic surgery were included in this study. The demographic and surgical characteristics are summarized in Table 1. The mean age of the participants was 26.6 ± 8.1 years, with a majority (76.8%) being women. Most patients (86.2%) underwent "mandible alone" surgery, while the remaining 13.8% received "bimaxillary" surgery. A total of 110 patients (60.8%) underwent BSSRO, followed by BSSRO + Gen (46 patients, 25.4%), Le Fort I + Gen (15 patients, 8.3%), Le Fort I + BSSRO (five patients, 2.8%), and Le Fort I + BSSRO + Gen (five patients, 2.8%). There was a significant difference in both operative time and total blood loss between these two groups.

**Table 1:** Summary of each related factor in this study P-values are calculated by two-tailed unpaired Student's t-test BMI, body mass index; PONV, postoperative nausea and vomiting; IPP, immediate postoperative pain; NRS, numeric rating scale; TIVA, total intravenous anesthesia

Variables		Overall (n = 181)	P-value
Gender	Male	42 (23.2%)	
	Female	139 (76.8%)	
Age (years)		26.6 ± 8.1	
BMI (kg/m^2^)	Mandible alone	21.6 ± 4.0	0.58
	Bimaxillary	21.2 ± 4.2	
Smoking	Yes	16 (8.8%)	
	No	165 (91.2%)	
PONV	Presence	89 (49.2%)	
	Absence	92 (50.8%)	
Surgical procedure	Mandible alone	156 (86.2%)	
	Bimaxillary	25 (13.8%)	
Operation time (minutes)	Mandible alone	143.4 ± 38.9	<0.0001>
	Bimaxillary	199.6 ± 46.1	
Total blood loss (g)	Mandible alone	52.3 ± 45.1	0.0034
	Bimaxillary	85.8 ± 52.9	
IPP (NRS)	Mandible alone	1.8 ± 2.6	0.035
	Bimaxillary	3.1 ± 3.4	
Anesthesia method	Inhalation anesthesia	161 (89.0%)	
	TIVA	20 (11.0%)	
Total amount of local anesthesia (mL)	Mandible alone	8.2 ± 1.5	<0.0001>
	Bimaxillary	11.2 ± 2.2	
Total amount of fentanyl (μg)	Mandible alone	186.5 ± 79.7	0.11
	Bimaxillary	214.0 ± 80.1	
Total amount of remifentanil (mg)	Mandible alone	2.4 ± 0.8	0.0005
	Bimaxillary	3.0 ± 0.9	

Immediate postoperative pain (IPP)

A total of 23.2% of the patients experienced "moderate to severe pain," while 76.8% reported "no pain to mild pain." The "bimaxillary" group showed higher IPP scores compared to the "mandible alone" group (Table 2).

**Table 2:** Statistical analysis between each independent variable and IPP *Pearson's chi-square test **Fisher's exact test IPP, immediate postoperative pain; BMI, body mass index; TIVA, total intravenous anesthesia; PONV, postoperative nausea and vomiting

Variables		No to mild (n = 139, 76.8%)	Moderate to severe (n = 42, 23.2%)	P-value
Gender	Male	32 (17.7%)	10 (5.5%)	0.92*
	Female	107 (59.1%)	32 (17.7%)	
Age (years)	<19	6 (3.3%)	5 (2.8%)	0.13**
	≥19	133 (73.5%)	37 (20.4%)	
BMI (kg/m^2^)	<19.56	39 (21.5%)	21 (11.6%)	0.01*
	≥19.56	100 (55.2%)	21 (11.6%)	
PONV	Presence	66 (36.5%)	23 (12.7%)	0.68*
	Absence	73 (40.3%)	19 (10.5%)	
Surgical procedure	Mandible alone	124 (68.5%)	32 (17.7%)	0.03*
	Bimaxillary	15 (8.3%)	10 (5.5%)	
Operation time (minutes)	<191	125 (69.1%)	31 (17.1%)	0.01*
	≥191	14 (7.7%)	11 (6.1%)	
Total blood loss (g)	<65	107 (59.1%)	25 (13.8%)	0.03*
	≥65	32 (17.7%)	17 (9.4%)	
Anesthesia method	Inhalation anesthesia	120 (66.3%)	41 (22.7%)	0.05**
	TIVA	19 (10.5%)	1 (0.6%)	
Total amount of local anesthesia (mL)	<9.9	113 (62.4%)	28 (15.5%)	0.05*
	≥9.9	26 (14.4%)	14 (7.7%)	
Total amount of fentanyl (μg)	<100	0 (0.0%)	3 (1.7%)	0.01**
	≥100	139 (76.8%)	39 (21.5%)	
Total amount of remifentanil (mg)	<2.7	103 (56.9%)	24 (13.3%)	0.04*
	≥2.7	36 (19.9%)	18 (9.9%)	

In multivariate analysis, significant predictors of IPP included low BMI (adjusted OR, 3.70; P = 0.0034) and the use of inhalation anesthesia (adjusted OR, 9.07; P = 0.049) (Table 3).

**Table 3:** Univariate and multivariate binary logistic regression analysis for IPP *P < 0.1 **Significant, P < 0.05 IPP, immediate postoperative pain; CI, confidence interval; OR, odds ratio; BMI, body mass index; TIVA, total intravenous anesthesia; PONV, postoperative nausea and vomiting; NA, not available

Variables	Univariate analysis			Multivariate analysis		
	Crude OR	95% CI	P-value	Adjusted OR	95% CI	P-value
Gender (female versus male)	0.96	0.41-2.16	0.92			
Age (<19 versus ≥19)	3.00	0.87-10.37	0.083*	2.61	0.58-11.76	0.21
BMI (<19.56 versus ≥19.56)	2.56	1.26-5.21	0.0092**	3.70	1.54-8.87	0.0034**
PONV (yes versus no)	1.34	0.67-2.68	0.41			
Surgical procedure (mandible alone versus bimaxillary)	0.39	0.16-0.94	0.037**	0.55	0.18-1.69	0.30
Operation time (<191 versus ≥191)	0.33	0.13-0.76	0.01**	0.56	0.17-1.80	0.33
Total blood loss (<65 versus ≥65)	0.44	0.21-0.91	0.028**	0.76	0.32-1.80	0.53
Anesthesia method (inhalation anesthesia versus TIVA)	6.49	0.84-50.02	0.073*	9.07	1.01-81.37	0.049**
Total amount of local anesthesia (<9.9 versus ≥9.9)	0.46	0.23-0.96	0.048**	0.60	0.23-1.59	0.31
Total amount of fentanyl (<100 versus ≥100)	NA	NA	0.99			
Total amount of remifentanil (<2.7 versus ≥2.7)	0.47	0.23-0.96	0.038**	0.43	0.15-1.26	0.12

Postoperative nausea and vomiting (PONV)

PONV was present in 49.2% of the patients. Significant differences were found between the "mandible alone" and "bimaxillary" groups regarding total blood loss and PONV incidence (Table 4).

**Table 4:** Statistical analysis between each independent variable and PONV *Pearson's chi-square test **Fisher's exact test PONV, postoperative nausea and vomiting; BMI, body mass index; TIVA, total intravenous anesthesia; IPP, immediate postoperative pain

Variables		With PONV (n = 89, 49.2%)	Without PONV (n = 92, 50.8%)	P-value
Gender	Male	17 (9.4%)	25 (13.8%)	0.20*
	Female	72 (39.8%)	67 (37.0%)	
Age (years)	<23	32 (17.7%)	38 (21.0%)	0.46*
	≥23	57 (31.5%)	54 (29.8%)	
BMI (kg/m^2^)	<18.6	24 (13.3%)	11 (6.1%)	0.01*
	≥18.6	65 (35.9%)	81 (44.7%)	
Smoking	Yes	5 (2.8%)	11 (6.1%)	0.19**
	No	84 (46.4%)	81 (44.7%)	
Surgical procedure	Mandible alone	76 (42.0%)	80 (44.2%)	0.76*
	Bimaxillary	13 (7.2%)	12 (6.6%)	
Operation time (minutes)	<159	58 (32.0%)	64 (35.4%)	0.53*
	≥159	31 (17.1%)	28 (15.5%)	
Total blood loss (g)	<40	33 (18.2%)	51 (28.2%)	0.01*
	≥40	56 (31.0%)	41 (22.6%)	
IPP	<3	57 (31.5%)	62 (34.3%)	0.64*
	≥3	32 (17.7%)	30 (16.6%)	
Anesthesia method	Inhalation anesthesia	82 (45.3%)	79 (43.6%)	0.18*
	TIVA	8 (3.9%)	13 (7.2%)	
Total amount of local anesthesia (mL)	<9.9	62 (34.2%)	79 (43.6%)	0.01*
	≥9.9	27 (14.9%)	13 (7.2%)	
Total amount of fentanyl (μg)	<100	2 (1.1%)	1 (0.5%)	0.62**
	≥100	87 (48.1%)	91 (50.3%)	
Total amount of remifentanil (mg)	<1.8	19 (10.5%)	11 (6.1%)	0.09*
	≥1.8	70 (38.7%)	81 (44.7%)	

In the multivariate analysis, low BMI (adjusted OR 2.80; P = 0.022), increased blood loss (adjusted OR 2.82; P = 0.005), and higher local anesthetic volume (adjusted OR 3.19; P = 0.016) were significant predictors of PONV (Table 5).

**Table 5:** Univariate and multivariate binary logistic regression analysis for PONV *P < 0.1 **Significant, P < 0.05 PONV, postoperative nausea and vomiting; CI, confidence interval; OR, odds ratio; BMI, body mass index; TIVA, total intravenous anesthesia; IPP, immediate postoperative pain.

Variables	Univariate analysis			Multivariate analysis		
	Crude OR	95%CI	P value	Adjusted OR	95%CI	P value
Gender (female vs male)	1.58	0.76-3.18	0.2	1.81	0.78-4.23	0.17
Age (＜23 vs ≧23)	0.8	0.44-1.45	0.46	0.7	0.34-1.42	0.32
BMI (＜18.6 vs ≧18.6)	2.72	1.24-5.96	0.013**	2.8	1.16-6.78	0.022**
Smoking (Yes vs No)	0.44	0.15-1.32	0.14	0.4	0.12-1.41	0.15
Surgical procedure (Mandible alone vs Bimaxillary)	0.88	0.38-2.04	0.76	2.37	0.77-7.30	0.13
Operation time (＜159 vs ≧159)	0.82	0.44-1.53	0.53	0.76	0.33-1.78	0.53
Total blood loss (≧40 vs <40)	2.11	1.16-3.83	0.014**	2.82	1.37-5.83	0.0050**
IPP (＜3 vs ≧3)	0.86	0.47-1.59	0.63	0.92	0.46-1.87	0.83
Anesthesia method (Inhalation anesthesia vs TIVA)	1.93	0.73-5.08	0.18	1.37	0.46-4.10	0.57
Total amount of local anesthesia (≧9.9 vs <9.9)	2.65	1.26-5.55	0.010**	3.19	1.25-8.16	0.016**
Total amount of fentanyl (＜100 vs ≧100)	2.09	0.19-23.5	0.55			
Total amount of remifentanil (＜1.8 vs ≧1.8)	2	0.89-4.49	0.093*	2.16	0.83-5.57	0.11

Relationship between IPP and PONV after orthognathic surgery

One of the most notable findings from this study was that low BMI was a common predictor for both IPP and PONV. Patients with a lower BMI were more susceptible to these postoperative complications. Additionally, the use of inhalation anesthesia was strongly associated with higher IPP, while the amount of intraoperative blood loss and the volume of local anesthesia were significant risk factors for PONV. Although both complications are frequent following orthognathic surgery, no significant direct correlation between IPP and PONV was identified in this study.

## Discussion

This study clarified the potential related factors of IPP and PONV after orthognathic surgery using binary logistic regression analysis. Multivariate binary logistic regression analysis of IPP and PONV highlighted BMI as a significant, common related factor. Patients with a low BMI were more susceptible to postoperative pain and the development of PONV. Inhalation anesthesia was identified as a significant related factor for IPP, while total blood loss exceeding 40 g and the use of more than 9.9 mL of local anesthesia were recognized as independent related factors for PONV.

Noxious stimuli resulting from tissue damage caused by osteotomies of the facial bones, as well as periosteal and muscle detachment during orthognathic surgery, are known to induce moderate to severe postoperative pain and swelling [5-8]. Then, these postoperative complications are the primary reasons for hospitalization following orthognathic surgery [5]. Studies on postoperative pain following orthognathic surgery are limited [4,5,19]. Postoperative pain is a subjective experience that may be influenced by various factors, including reporting bias, preoperative pain, anxiety, mood, and the type of surgery [21-23]. Commonly reported related factors include age, gender, and the type of surgery, and in the case of orthognathic surgery, a relationship with PONV has also been noted [5,19,22,24-26]. Although the present study examined the correlation between PONV and IPP, no significant relevance was identified. In the present study, inhalational anesthesia was identified as a significant associated factor of IPP through multivariate analysis. Although several studies have explored the relationship between anesthesia techniques and postoperative pain, no reports have focused on orthognathic surgery. Previous studies have suggested that propofol total intravenous anesthesia (TIVA) may reduce postoperative pain and decrease the requirement for analgesics compared to inhalational anesthesia, while some studies have reported no significant superiority of propofol TIVA [27-31]. Peng et al. conducted a systematic review and meta-analysis on postoperative pain management following propofol versus inhalational anesthesia [32]. Their findings indicated that propofol anesthesia was associated with lower postoperative pain intensity, reduced opioid consumption, and prolonged time to the first administration of analgesics compared to inhalational anesthesia. While inhalational anesthesia inhibits the transmission of sensory afferent stimuli to the central nervous system [33,34], their use at low concentrations, such as 0.1 minimum alveolar concentration (MAC), may induce hyperalgesia and exacerbate postoperative pain perception [35]. In contrast, propofol has demonstrated short-acting analgesic properties and a tendency to suppress hyperalgesia and allodynia in healthy individuals [36]. Given the methodological inconsistencies in previous studies, further large-scale randomized controlled trials, specifically addressing disease-specific populations, are warranted to provide more definitive conclusions.

PONV is one of the most frequent and distressing complications following various inpatient and outpatient surgeries. PONV following orthognathic surgery is multifactorial, with previous studies identifying gender, age, surgical technique (single-jaw osteotomy alone or bimaxillary osteotomy), the duration of surgery, and blood loss as contributing factors [18,19,37]. In a large retrospective study, Silva et al. reported that 40% of orthognathic surgery patients experienced PONV within the first 24 hours postoperatively, with a notably higher incidence of 56% in patients who underwent bimaxillary osteotomy [37]. Phillips et al. found that 67% of patients experienced nausea, and 27% experienced vomiting after orthognathic surgery [18]. While patients undergoing bimaxillary osteotomy had higher rates of PONV compared to those undergoing single-jaw surgery, these differences were not statistically significant. In the present study, although the incidence of PONV was higher after the bimaxillary group, no statistically significant difference was observed in comparison to the mandible-alone group.

In the present study, multivariate analysis identified the total amount of local anesthesia and total blood loss as significant related factors of PONV. To date, no studies have specifically focused on the correlation between the amount of local anesthesia during orthognathic surgery and PONV. The relationship between local anesthesia and PONV has a lack of evidence. There is no compelling evidence to suggest that using a specific local anesthesia significantly influences the occurrence of PONV [38]. A comparative study between lidocaine and procaine found no statistically significant difference in PONV incidence [39]. However, some reports indicate that the addition of epinephrine to local anesthesia may contribute to the development of PONV [40,41]. Previous studies have demonstrated that combining procaine with epinephrine increases the incidence of PONV from 10% with procaine alone to 30% with the combination [40]. It has been hypothesized that epinephrine may promote the release of serotonin or stimulate the chemoreceptor trigger zone via sympathetic beta-adrenergic receptors [42,43], though the precise mechanism remains to be fully elucidated.

Bleeding is a significant complication in orthognathic surgery, occurring primarily during maxillary down fraction following Le Fort I osteotomy or during separation at the pterygomaxillary junction [44,45]. It is widely believed that swallowing surgical fluids, particularly blood, during surgery contributes to the occurrence of PONV [46]. Recently, Hoshijima et al. demonstrated through machine learning modeling that the volume of intraoperative blood loss is a significant related factor for PONV [20]. In oral and maxillofacial surgery, throat packs have traditionally been placed in the pharyngeal cavity to prevent the inflow of surgical fluids, especially blood. However, there is insufficient evidence to confirm whether throat packs effectively serve as a physical barrier against surgical fluids and whether throat packs can induce postoperative sore throat and dysphagia [9]. Powell et al. reported that there was no significant difference in the gastric contents aspirated via nasogastric tube between patients with and without throat packs [47]. Oliveira De Jesus et al. [48] and Wang and Zhang [49] reported that gastric aspiration was beneficial in preventing PONV. In the present study, a nasogastric tube was inserted in the "bimaxillary" group, but there was no significant difference in the incidence of PONV compared to the "mandible alone" group.

A relationship between postoperative pain and PONV following orthognathic surgery has been reported, though it was not identified as a significant related factor in the present study [19]. A common finding across IPP and PONV is that a low BMI serves as a shared significant related factor for both IPP and PONV. Regarding BMI and orthognathic surgery, Silva et al. [37] and Phillips et al. [18] reported that obese patients are less prone to developing PONV, though the exact reason for this remains unclear. Generally, in patients with low BMI, the volume of drug distribution tends to decrease, and there is less accumulation of drugs in adipose tissue, leading to higher plasma concentrations of medications and potentially stronger pharmacological effects [50]. In patients with low BMI, it may be conceivable that the effects of anesthetics and epinephrine, which were identified as related factors for both IPP and PONV, are more pronounced, resulting in increased pain sensitivity and a higher likelihood of developing PONV.

This study has a few limitations. First, it is a single-center retrospective cohort study with a relatively small sample size. Second, the "bimaxillary" group was notably smaller than the "mandible alone" group. Nonetheless, few previous studies have simultaneously analyzed both IPP and PONV following orthognathic surgery.

## Conclusions

This study identified the key determinants associated with IPP and PONV following orthognathic surgery. Notably, BMI was found to be a significant common factor for both IPP and PONV, with lower-BMI patients being more susceptible to these complications. Inhalation anesthesia was associated with IPP, while higher blood loss and the amount of local anesthesia were identified as independent factors related to PONV. These findings provide new insights that can contribute to improving preoperative assessments and anesthesia management to enhance patient comfort after orthognathic surgery. Further large-scale, multicenter studies are needed to explore additional potential factors influencing the occurrence of IPP and PONV and to establish more comprehensive postoperative care guidelines. The results of this study may contribute significantly to improving patient care in the context of orthognathic surgery.
