# Correction: Key Determinants of Immediate Postoperative Pain, Nausea, and Vomiting in Orthognathic Surgery: Insights From a Retrospective Cohort Study

**DOI:** 10.7759/cureus.c222

**Published:** 2025-05-09

**Authors:** Shogo Kikuta, Sho Imai, Nodoka Nagae, Katsuhisa Matsuo, Kiyosato Hino, Yushi Abe, Jingo Kusukawa

**Affiliations:** 1 Dental and Oral Medical Center, Kurume University School of Medicine, Kurume, JPN

This article has been corrected as the original odds ratios and confidence intervals were incorrectly calculated. The corrections are as follows:

The abstract previously stated “higher blood loss and increased local anesthesia volumes were associated with PONV (adjusted OR, 0.35 and 0.31; P = 0.005 and 0.016, respectively).” This has been corrected to “higher blood loss and increased local anesthesia volumes were associated with PONV (adjusted OR, 2.82 and 3.19; P = 0.005 and 0.016, respectively).”

The results section previously stated “In the multivariate analysis, low BMI (adjusted OR, 2.80; P = 0.022), increased blood loss (adjusted OR, 0.35; P = 0.005), and higher amounts of local anesthesia (adjusted OR, 0.31; P = 0.016) were significant predictors of PONV (Table 5).” The corrected version is “In the multivariate analysis, low BMI (adjusted OR 2.80; P = 0.022), increased blood loss (adjusted OR 2.82; P = 0.005), and higher local anesthetic volume (adjusted OR 3.19; P = 0.016) were significant predictors of PONV (Table 5).”

In Table 5, the row ‘Total blood loss (≧40 vs <40)’ had previously reported univariate analysis crude OR at 0.47, univariate analysis 95% CI at 0.26-0.86, adjusted OR at 0.35, and 95% CI at 0.17-0.73. This has been corrected as univariate analysis crude OR at 2.11, univariate analysis 95% CI at 1.16-3.83, multivariate analysis adjusted OR at 2.82, and multivariate analysis 95% CI at 1.37-5.83.

In Table 5, the row ‘Total amount of local anesthesia (≧9.9 vs <9.9)’ had previously reported univariate analysis crude OR at 0.38, univariate analysis 95% CI at 0.18-0.79, multivariate analysis adjusted OR at 0.31, and multivariate analysis 95% CI at 0.33-1.78. This has been corrected as univariate analysis crude OR at 2.65, univariate analysis 95% CI at 1.26-5.55, multivariate analysis adjusted OR at 3.19, and multivariate analysis 95% CI at 1.25-8.16.

